# DNA Fragmentation Analysis in Human Sperm—Technical Instructions to Prevent False Positives and Negatives in Angle-Modulated Two-Dimensional Single-Cell Pulsed-Field Gel Electrophoresis

**DOI:** 10.3390/genes17030319

**Published:** 2026-03-16

**Authors:** Satoru Kaneko, Yukako Kuroda, Yuki Okada

**Affiliations:** 1 Laboratory of Pathology and Development, Institute for Quantitative Biosciences, The University of Tokyo, 1-1-1 Yayoi, Bunkyo, Tokyo 113-0032, Japan; ytokada@iqb.u-tokyo.ac.jp; 2 Sperm-Semen-Epididymis-Testis (SSET) Clinic, 1-5 Kanda-Iwamoto, Chiyoda, Tokyo 101-0033, Japan; 3 Department of Obstetrics and Gynecology, Fukushima Medical University, Hikarigaoka, Fukushima 960-1295, Japan; kuroda-y@fmu.ac.jp; 4 KURODA International Medical Reproduction, 2-22-1 Shinkawa, Chuo, Tokyo 104-0033, Japan

**Keywords:** human sperm, single-cell pulsed-field gel electrophoresis, male infertility, DNA fragmentation, reactive oxygen species, telomere, false-positive, false-negative

## Abstract

Over the past two decades, numerous studies have examined the etiological significance of DNA fragmentation in human sperm using methods such as the comet assay (CA), the sperm chromatin structure assay, the sperm chromatin dispersion assay, and the TUNEL assay. We developed single-cell pulsed-field gel electrophoresis techniques, including one-dimensional (1D-SCPFGE) and angle-modulated two-dimensional (2D-SCPFGE), to detect early signs of naturally occurring DNA fragmentation. Comparative studies using purified human sperm with and without DNA fragmentation revealed some technical limitations in the conventional methods. This technical review outlines the procedures to ensure the quantitative performance of SCPFGE: (1) The mass of naked DNA was prepared through simultaneous in-gel swelling and proteolysis, which are highly sensitive to chemical and physical factors. Notably, these processes are vulnerable to reactive oxygen species (ROS). We developed the anti-ROS SCPFGE system to prevent artifactual cleavages. (2) 1D-SCPFGE discharges long-chain fibers from the origin, separating fibrous and granular segments beyond the tips of the fibers. (3) During continuous electrophoresis after 150° rotation (2D-SCPFGE-0-150), long-chain fibers unexpectedly extended diagonally backward from the origin, with long fibrous segments pulled out from a bundle that extended during the first electrophoresis, indicating some fibrous segments were embedded within the long-chain fibers. Even when SCPFGE was employed, one-directional current led to false negatives. (4) 2D-SCPFGE with angle rotation is currently the most sensitive imaging method for single-nuclear DNA fibers. However, without knowing the size of DNA fragments, it remains a semi-quantitative analysis. (5) To prevent artifactual DNA cleavage caused by ice crystals, low-temperature liquid storage is recommended. (6) The in-gel proteolyzed naked DNA is suitable as a substrate for chemical and enzymatic DNA cleavage analyses.

## 1. Introduction

Each individual is born with a few novel genetic changes, known as de novo mutations, which occur either during gamete formation or embryogenesis [1]. Both DNA repair and apoptotic mechanisms effectively remove damaged germ cells [2], but these elimination capacities decline during late spermatogenesis, allowing DNA lesions to accumulate in sperm [2,3]. The presence of DNA damage in sperm has been experimentally proven to cause embryonic developmental defects and congenital abnormalities in the next generation [4], unless it is repaired by maternal factors after fertilization [5]. Due to the lack of homologous templates, zygotic repair of paternal double-strand breaks (DSBs) relies on non-homologous repair mechanisms, which are considered error-prone. This largely explains why most de novo mutations, including copy number variations in offspring, originate from the paternal side [6]. In somatic cells, single-strand breaks (SSBs) are the most common DNA lesions, with tens of thousands occurring daily in each cell [7]. Base excision repair is a primary pathway for fixing harmful DNA lesions [8], including non-bulky DNA adducts, apurinic/apyrimidinic (AP) sites [9], and SSBs. If these lesions are not repaired or are repaired incorrectly, they can threaten genetic integrity by converting into DSBs during DNA replication. DSBs are the most challenging DNA lesions to repair, and the critical number of DSBs in a nucleus may be very low [10,11]. The quantity of DSBs does not directly correlate with the occurrence of harmful DNA lesions, since exceeding a critical threshold can lead to fertilization failure or pregnancy loss. Detecting early signs of DNA fragmentation is essential for diagnostic use in clinical assisted reproductive technology (ART). Given their relative frequencies, single-nuclear DSBs and SSBs, including AP sites, are best analyzed together.

Numerous studies have examined the effects of DNA damage on fertilization, post-implantation embryo development [12], and sperm-derived congenital anomalies in ART [13]. Over the past two decades, nonspecific single-nuclear DNA damage in human sperm has been studied using various assays such as the comet assay (CA) [14,15], sperm chromatin structure assay (SCSA) [16,17], sperm chromatin dispersion test [18,19], and terminal deoxynucleotidyl transferase-mediated dUTP nick-end labeling assay [20]. We prepared human sperm with and without DNA fragmentation and analyzed their cellular properties [21,22,23,24,25]. Comparative studies with these two extremes indicated that the above-mentioned techniques failed the initial step of qualitative validation due to their technical limitations [21,22,23,24,25].

Conventional submarine gel electrophoresis (SGE) is used in the CA, and the level of DNA damage is assessed by counting the number of granular fragments released from the origin, known as the comet tail [14,15]. We developed one-dimensional single-cell pulsed-field gel electrophoresis (1D-SCPFGE) to overcome the limitations of SGE [26,27]. The in-gel proteolyzed DNA mass released long-chain fibers from the origin, and fibrous and granular segments separated beyond the tips of these fibers. Later, we introduced a new angle-modulated two-dimensional single-cell pulsed-field gel electrophoresis (2D-SCPFGE) [25,28]. After continuing electrophoresis following a 150° rotation, long-chain fibers unexpectedly extended at an angle backward from the origin, and fibrous segments were pulled out from a bundle of long-chain fibers that extended during the first electrophoresis. Even when using pulsed-field gel electrophoresis, employing only one-dimensional current sometimes led to false negatives in DNA fragmentation analysis [25,28].

We developed the following technical competencies to reduce false positives and negatives: (1) purification of motile sperm without DNA fragmentation and immotile sperm with end-stage fragmentation from human semen, for use as comparative standards and test objects; (2) low-temperature liquid storage in a non-aqueous solvent to preserve the integrity of DNA sequences; (3) spreading and adherence of sperm to a glass slide; (4) embedding sperm into a thin agarose film; (5) in-gel swelling of the tightly packed nucleus; (6) in-gel proteolysis of nucleoproteins to prepare the naked DNA fibers; (7) designing a round electrophoresis tank with three electrode pairs; (8) preparing the antioxidative electrophoresis buffer; (9) developing angle-modulated 2D-SCPFGE for separating long fibrous segments from intact fibers; (10) 2D alignment of single-nuclear naked DNA fibers; (11) creating post-electrophoretic fluorescent staining and anti-DNA fragmentation additives to protect DNA sequences during blue light excitation; and (12) processing images of the region of interest (ROI).

Although 2D-SCPFGE was developed as a tool for DNA fragmentation analysis, with angle rotation providing various 2D alignments of single-nuclear DNA fibers, it also opens up new horizons in the vast field of DNA science, as discussed in this review.

## 2. Observation of DNA in Macro- and Micro-PFGE

Agarose can be used to create large-pore, thermoplastic gels composed of bundles of polysaccharide chains linked by hydrogen bonds. SGE with a one-directional current is the most common form of macroscopic gel electrophoresis used for DNA analysis. Its main components are the proteolysis of the cell mass, subsequent deproteination (such as with spin columns containing glass-fiber filters), and the migration of naked DNA through several centimeters of the gel. Because the phosphate ions in the DNA are fully dissociated, the negatively charged DNA moves toward the cathode, regardless of the pH of the electrophoresis buffer, at a rate inversely related to DNA size. The composition and ionic strength of the buffers, along with voltage–current characteristics, affect the electrophoretic behavior of DNA. Running known DNA size markers simultaneously allows for comparison, helping to determine the size of DNA fragments in base pairs based on their electrophoretic mobility.

Macro-PFGE, developed by Schwartz and Cantor in 1984 [29], allows the determination of DNA fragment sizes of up to 10 Mb. The gel size is similar to that used in normal SGE; however, the key feature of macro-PFGE is that the voltage direction is periodically reversed, causing large DNA molecules to migrate in a zigzag pattern through the agarose pores [30,31]. Since long DNA is very fragile and prone to shearing, the total amount of pure cultured prokaryotes is embedded in an agarose plug and then proteolyzed. Additionally, pre-electrophoretic downsizing with restriction endonucleases is necessary for segments of prokaryotic DNA to migrate effectively in macro-PFGE. Currently, bacterial DNA fingerprinting is the primary application of macro-PFGE, while its use for eukaryotes is limited to yeast [29].

Single-cell micro-PFGE [25,26,27,28] differs significantly from the two aforementioned macroscopic methods. The test objects are free cells or cells dispersed from eukaryotes. These are either suspended in melted agarose to form a thin film on a glass slide or spread on the slide using automatic centrifugal smearing, then embedded in agarose to create thin films. After in-gel proteolysis, the mixture of intact chromosomal DNA and fragments of various sizes is electrophoresed without being cut down by restriction endonucleases. The electrophoretic profiles are visualized using fluorescent microscopy.

## 3. Preparation of Comparative Standards for DNA Fragmentation Analyses

We previously prepared human motile sperm without DNA fragmentation and immotile sperm with end-stage DNA fragmentation as comparative standards for DNA fragmentation analyses, as detailed below [25,28]. In clinical ART, the former can be used for in vitro fertilization as well as intra-cytoplasmic sperm injection (ICSI).

### 3.1. Equilibrium Sedimentation in OptiPrep

Sperm concentration and motility were assessed following the World Health Organization reference manual [32]. We diluted semen samples three times with 20 mmol/L HEPES-buffered Hanks’ solution (pH 7.4; hereafter called “Hanks”). OptiPrep density gradient medium (Axis Shield, San Jose, CA, USA) was prepared to be isotonic using 20 mmol/L HEPES-NaOH (pH 7.4), Hanks’ powder, and 2.0 mg/mL human serum albumin (density: 1.17 g/mL; hereafter called “OP”). The suspension was layered on 0.5 mL of OP and centrifuged at 600× *g* for 10 min. The precipitate and interphase layer were resuspended to 1.0 mL. This was layered on 0.5 mL of OP and ultracentrifuged at 10,000× *g* for 10 min to separate the interphase layer from the sediment [25,28].

### 3.2. Differential-Velocity Sedimentation in Percoll

The interphase layer and sediment were separated, diluted multiple times with Hanks, and centrifuged in a 90% Percoll (GE Healthcare, Chicago, IL, USA) density gradient as previously described [21,22,25,28]. The 90% Percoll was made isotonic with 20 mmol/L HEPES-NaOH (pH 7.4), Hanks’ powder, and 2.0 mg/mL human serum albumin (density: 1.12 g/mL; hereafter called “Percoll”). Then, 5.0 mL Percoll was placed in a 15 mL conical test tube, and 1.0 mL Hanks was added. This mixture was gently mixed to form a linear density gradient by 10 revolutions at a 30° angle. The sperm suspension was layered on the gradient and centrifuged with a swing-out rotor at 400× *g* for 30 min. Motile sperm without DNA fragmentation were collected from the interface layer of OP or the Percoll sediment. Hanks (1.0 mL) was layered on the Percoll sediment (0.2 mL) and incubated for 60 min at room temperature. Sperm that swam to the upper half of the Hanks layer were recovered. Immotile sperm with advanced DNA fragmentation were obtained from the sediment of OP or the intermediate Percoll layer [21,22,25,28]. The motile sperm are called “purified sperm” (PS), and the immotile sperm are called “denatured sperm” (DS). These two groups differ significantly in cellular features, reflecting sperm that have not yet undergone apoptosis and those that have already undergone apoptosis and denaturation, respectively [21,22,25,28]. PS was prepared from about a dozen semen samples; the sample with the lowest DNA fragmentation rate determined by 1D-SCPFGE was used as the standard. DS were prepared from the same semen samples. Both PS and DS were stored in a low-temperature preservation medium (see Section 5.5).

## 4. Lessons Learned from Conventional Analytical Methods

**Figure 1:**
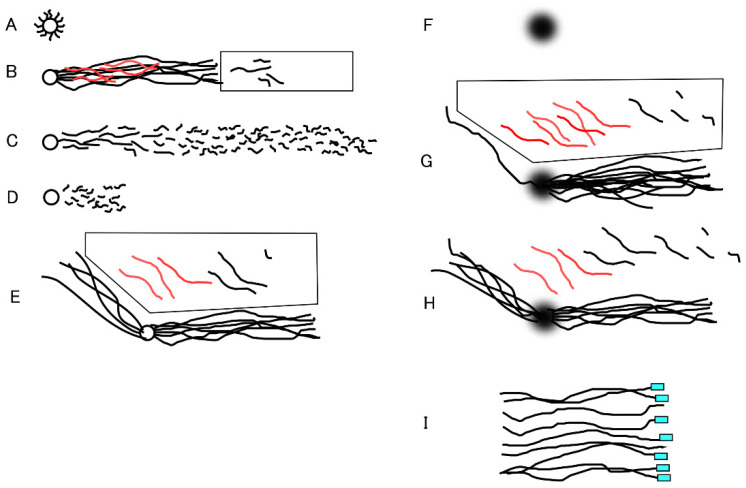
Schematic illustration of long-chain fibers and fibrous and granular segments in 1D- and 2D-SCPFGE. (**A**) In-gel proteolyzed DNA mass without in-gel swelling (former protocol). (**B**) 1D-SCPFGE linearly arranged long-chain fibers and segments. The rectangular window beyond the tips of the elongated long-chain fibers shows ROI. If no segments were found in the ROI, the former protocol was deemed to be sequentially intact. Fibrous segments (the red lines) remained entangled in the bundle of long-chain fibers, causing a false negative. (**C**) End-stage fragmentation. (**D**) CA lacks in-gel proteolysis and SPFGE; it cannot elongate the long-chain fibers, and smaller granular segments are observed compared with those in (**C**). (**E**) The original 2D-SCPFGE-0-150 without in-gel swelling. All the segments separated beyond the tips of the elongated long-chain fibers in the first electrophoresis were oriented obliquely backward and assembled in the polygonal window. (**F**) DNA mass after simultaneous in-gel swelling and proteolysis. (**G**) In the first electrophoresis, the swollen DNA mass discharged a greater number of long-chain fibers than in (**E**). (**H**) Evaporation of some water in the gel film hindered all efforts; the number of fibers discharged obliquely backward increased again. Therefore, all processes should be performed in the moisture chamber to obtain the profile in (**G**). (**I**) Expected profile of telomeric analysis. The image shows the tips of a set of single-nuclear long-chain fibers aligned by means of 2D-SCPFGE-(-75)-0. The telomeric regions are labeled with PNA-FISH. The fluorescent signal at the tips represents the telomeric end, and those without signals represent cut ends.

Before newly developed analytical methods are used for clinical diagnoses, they must pass multiple validation steps using comparative standards. Conventional DNA damage analyses cannot meet the initial step of quantitative validation because of their underlying principles and technical limitations. Figure 1 illustrates the development process of 1D- and 2D-SCPFGE. This section explains the issues with SCSA and CA.

### 4.1. SCSA Overlooks Interference of Nucleoproteins

SCSA employs a simple bisection principle in which the intercalation of monomeric acridine orange (AO) into double-stranded DNA or the adsorption of oligomeric AO to single-stranded DNA produces green or red fluorescence, respectively [16,17]. Several experiments have demonstrated that AO cannot detect DNA damage in human sperm: AO staining cannot distinguish PS from DS under a microscope or via flow cytometry; the nucleus and cytoplasm of lymphocytes emit green and red fluorescence, respectively; and in-gel tryptic digestion removes the red fluorescence [21,25]. These phenomena suggest that green fluorescence results from intercalated AO, while red fluorescence comes from AO adsorbed to nucleoproteins rather than single-stranded DNA. DNA fluorescent probes often interact with various intercellular materials besides DNA, and the fact that SCSA does not account for the interference of nucleoproteins [21,25] emphasizes the importance of quantitative validation using PS and DS.

A technical issue in SCSA is that unseparated human semen is used as the test specimen [16,17]. Even in normozoospermia, more than half of the sperm in a semen sample are already denatured, showing end-stage fragmentation [23]. As mentioned in Section 3, PS is a preferable alternative in clinical ART. The goal of DNA damage analyses is to detect early signs in the separated PS; unseparated semen is not eligible.

### 4.2. Lack of In-Gel Proteolysis and SCPFGE Impairs the Sensitivity of the Neutral and Alkaline CA

In neutral CA, a high-salt extraction of nucleoproteins and conventional SGE is used, and the level of DNA damage is assessed by counting the number of granular segments (the comet tail) [14,15]. High-salt extraction and discharged granular segments from DS (Figure 1D) but did not elongate long-chain fibers from PS. 1D-SCPFGE pulled out long-chain fibers (Figure 1B) from the in-gel proteolyzed PS (Figure 1A). In-gel proteolysis of DS results in a dramatic increase in granular segments (Figure 1C) [22]. 1D-SCPFGE tracks DNA fragmentation in human sperm; initially, a few large fibrous segments appear beyond the front of a bundle of elongated long-chain fibers, and the process continues until almost all DNA fibers are shredded into granular segments [26,27]. The neutral CA can only be used to detect the final stage of fragmentation.

Human sperm nucleoproteins consist of protamines [33,34], histones [35], condensins [36], and cohesins [36]. The DNA–nucleoprotein complex is attached to the nuclear membrane through the nuclear matrix [37,38]. Although high-salt solutions are commonly used to extract protamines [39], some nucleoproteins remain fixed to both intact DNA fibers and segments. Only a portion of the granular segments escapes fixation and is released as the comet tail. Comparative experiments between PS and DS highlighted the importance of in-gel proteolysis and SCPFGE, as neither the SCSA nor the neutral CA accounts for nucleoprotein interference, and only naked DNA can be analyzed via electrophoresis.

Alkaline-labile sites (ALSs), such as AP sites [8,9], cause DNA strand cleavage at high pH. The alkaline CA, where DNA is treated with 300 mmol/L NaOH [14,15], induces DSBs from SSBs and at ALSs. Alkaline-based SSB assays have a high risk of overestimating damage. When PS is treated with 30 mmol/L NaOH, 1D-SCPFGE with in-gel proteolysis shows the release of more granular segments than neutral CA with high-salt extraction and SGE [22]. Some nucleoproteins resist high alkalinity, remaining attached to new granular segments through alkaline hydrolysis, which can hinder their electrophoretic migration. Lack of in-gel proteolysis can lead to false-negative results in both neutral and alkaline CAs. Due to space constraints, this review highlights the technical limitations of SCSA, CA, the sperm chromatin dispersion test [22], and the terminal deoxynucleotidyl transferase-mediated dUTP nick-end labeling assay [25]. They emphasize the importance of using comparative standards, calibration curves, appropriate sensitivity, and eligibility criteria for test subjects to validate the quantitative performance of analytical methods in this field.

## 5. Technical Specifications for 1D- and 2D-SCPFGE

The operating procedures for 1D- and 2D-SCPFGE are similar. The only difference is that the latter involves one or more angle rotations after the initial electrophoresis. This section outlines the technical specifications of SCPFGE.

### 5.1. Adherence of Agarose Film to a Glass Surface

For agarose adhesion, we used commercially available MAS-coated glass slides (Matsunami, Tokyo, Japan), on which amino residues were introduced through chemical surface modification to prevent histological sections from detaching from the slide. The agarose used for embedding was bound to the slide at an acidic pH. As described in Section 5.3, we changed the pH of the agarose for embedding from 4.7 (acetate buffer) [22,25,26,27] to 9.0 (Na_2_CO_3_-NaHCO_3_). Although agarose interacts with the ionized amino residues via hydrogen bonds at an acidic pH, it does not interact with non-ionic residues at pH 9.0.

The surface of the glass slide (2.5 × 6.0 cm area) was coated with 30 μL of 1.0% low-melting-temperature agarose (agarose GB; Nippon Gene, Tokyo, Japan; melts at around 80 °C and gels at around 25 °C). Once dehydrated, agarose firmly attaches to the glass surface, and the agarose used for embedding adheres through hydroxyl–hydroxyl hydrogen bonds.

### 5.2. Equipment for SCPFGE

**Figure 2:**
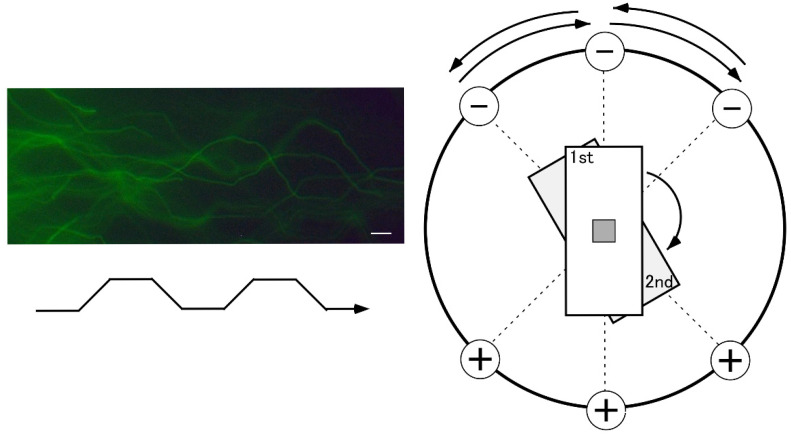
Schematic illustration of the round electrophoresis tank and rotation of the rack for 2D-SCPFGE-0-15. Centrifugal auto-smearing was used to adhere human sperm to a 5 × 5 mm area of the agarose-coated glass slide. The critical technical aspect is the precise placement of this area exactly on the intersection point of the three currents. Positioning errors cause deformation of the electrophoretic profiles. The arrows indicate the sequential flow of the active current during SVPFGE. The long-chain fibers migrate in S-shaped curves depending on the switching intervals. Scale bars represent 10 µm. All the color photographs in in this and the following figures display the fluorescent profiles of DNA stained with SYBR Gold.

Figure 2 shows a schematic of the round electrophoresis tank used for 2D-SCPFGE, featuring three pairs of electrodes spaced 45 degrees apart. The apparatus should be placed on a stable, level surface. Five glass slides with gel films are stacked vertically in a rack with a pivot for rotation. The embedded DNA should be precisely positioned on the pivot at the intersection of the three currents. Electrophoresis is conducted at a constant voltage (3.5 V/cm), with 4.0 s switching intervals following this sequence: left, center, and right, then reversing. We developed an in-house time-switching controller. The electrophoresis buffer is at least 4 cm deep to immerse all five slides. A combination of weak organic electrolytes (see Section 5.6) was designed to prevent overcurrent to the power supply. High-magnification images in Figure 2 show the S-shaped tracks of long-chain fibers. This shape varies depending on the voltage, switching interval, ionic strength of the buffer, and pore size of the agarose.

### 5.3. Simultaneous In-Gel Swelling and Proteolysis

**Figure 3:**
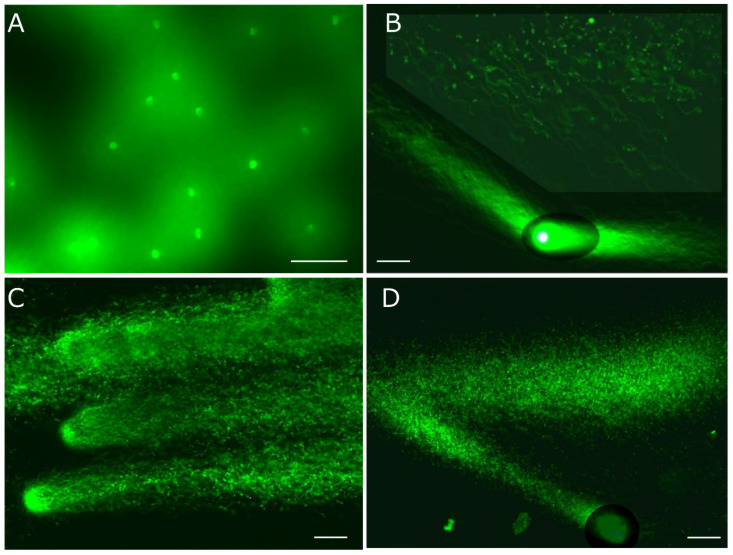
The former protocol of D-SCPFGE-0-150 without in-gel swelling. (**A**) After embedding, the agarose was immediately chilled on ice for 5 min, followed by in-gel proteolysis at 45 °C for 10 min. The tightly packed DNA mass appeared as a small, bright core. (**B**) 2D-SCPFGE-0-150 shows elongated long-chain fibers in two directions, while the bright core remained at the origin. (**C**) 1D-SCPFGE profile of the end-stage fragmentation in DS. (**D**) 2D-SCPFGE profile of the end-stage fragmentation in DS. Scale bars represent 50 µm.

1D-SCPFGE with in-gel proteolysis results in a linear arrangement of the elongated long-chain fibers and the segments (Figure 1C) [22,26,27]. Former protocols for SCPFGE solidified the agarose immediately after embedding to prevent in-gel proteolysis, and the tightly packed DNA mass remained visible as a small, bright core (Figure 3A). We developed 2D-SCPFGE-0-150 (6/4 min) [28], in which the gel was rotated by 150 degrees after the first electrophoresis. All the segments that were previously separated beyond the tips of the elongated long-chain fibers became oriented obliquely backward and assembled in the shooting range of a charge-coupled device camera with 200× magnification (Figure 1E and Figure 3B). The second electrophoresis produced unexpected results: additional long-chain fibers were discharged obliquely backward from the origin and spread out in a fan-like shape (Figure 1E) [28]. The long fibrous segments were drawn out from a bundle of elongated fibers to the inner angle of the fan (Figure 3B). The bright core was still visible at the origin. Figure 3C,D show the profiles of 1D- and 2D-SCPFGE of DS; almost all the fibers had already been shredded into granular segments, and no bright core was found at the origin. 1D-SCPFGE is sufficiently sensitive and quite convenient for observing the advanced and final stages of fragmentation.

**Figure 4:**
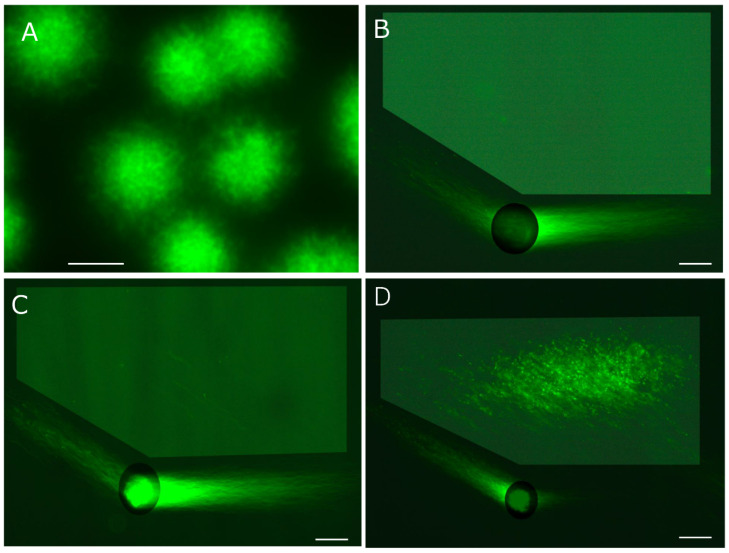
The updated protocol of 2D-SCPFGE-0-150 with in-gel swelling. (**A**) The agarose was kept at 37 °C for 5 min before chilling on ice, which caused swelling of the sperm heads without free diffusion of the fibers. The bright core disappeared. The images labeled (**B**–**D**) show typical profiles of DNA fragmentation. (**B**) Compared to Figure 3B, more long-chain fibers were discharged from the swelling mass during the first electrophoresis, and those discharged obliquely backward at the second run were reduced. This represents the typical profile of intact sperm, with no segments detected in the ROI. (**C**) The typical profile of early-stage fragmentation shows a few long fibrous segments in the ROI. (**D**) The typical profile of advanced-stage fragmentation displays various sizes of fibrous and granular segments in the ROI. Scale bars represent 50 µm.

To maximize the number of long-chain fibers drawn in two directions, the tightly packed DNA mass was swollen through simultaneous in-gel swelling and proteolysis (Figure 1F and Figure 4A). Human sperm (2 × 10^2^) were adhered to the agarose-coated glass slide by centrifugal auto-smearing. Specifically, 0.5 mL of 0.4% agarose GB (containing 0.1 M Na_2_CO_3_-NaHCO_3_, pH 9.0, 0.02% SDS, 1.0 mmol/L EDTA) was melted at 85 °C for 10 min, then 10 μL of proteinase K (recombinant, 20 mg/mL; Thermo Fisher Scientific, Waltham, MA, USA), 0.5 mol/L DTT in 0.1 mol/L acetate buffer (pH 4.7), and 1.0 mol/L edaravone (3-methyl-1-phenyl-2-pyrazolin-5-one; ED) in DMSO were added at 42 °C just before use (final concentration: 0.38% agarose). An aliquot of 60 μL of this solution was placed on the sperm and covered with a glass slip (2.4 × 2.4 cm) for a thickness of 100 μm. The in-gel swelling (37 °C), chilling on ice (4 °C), and proteolysis (45 °C), described below, were performed in a moisture chamber. The pharmacological properties of ED are discussed in detail in Section 5.5, Section 5.6 and Section 5.7.

The agarose was immediately chilled on ice for 5 min, followed by in-gel proteolysis at 45 °C for 10 min. The DNA mass was enclosed in a small, bright core (corresponding to Figure 3A). Although 2D-SCPFGE-0-150 yielded the elongation of long-chain fibers in two directions, some of these fibers remained within the small, bright core at the origin (corresponding to Figure 3B). When the agarose was incubated at 37 °C for 5 min on a hot plate before chilling on ice, the semisolid gel allowed swelling of the sperm heads without free diffusion of the fibers (Figure 4A). The increased void space facilitated the permeation of proteinase K. After swelling, the first electrophoresis resulted in the discharge of more long-chain DNA from the swelling mass, while the fibers discharged at an angle during the second run were significantly fewer (Figure 1G and Figure 4B–D). This profile suggests that swelling untangled the DNA fibers, making their removal more efficient. After brightening the ROI, no segments were observed in the inner angle of the fan, which was considered intact based on our tentative definition (see Section 5.7 and Section 7) (Figure 4B). Figure 4C,D show typical profiles of early and advanced fragmentation stages, respectively. If the agarose was incubated at 42 °C for 10 min before chilling, the sperm heads melted, and DNA fibers diffused freely in the liquefied agarose.

The most critical factor for simultaneous in-gel swelling and proteolysis is changing the pH of the agarose used for embedding from 4.7 to 9.0. The acid dissociation constants of the two thiol residues in DTT are 9.2 and 10.1, which is why the inter- and intra-disulfide bonds among protamines must be reduced at a pH above 9. The optimal pH for proteinase K is 8 or higher [40]. The guanidino residues of arginine in protamines form ionic bonds with DNA phosphates, and the presence of DTT and other sulfate-containing compounds like SDS can competitively dissociate the DNA–protamine complex at pH 9 or above, leading to swelling of the sperm head. SDS showed the strongest swelling activity among the organic compounds tested. Only 0.005% SDS induces head swelling in the suspension, while the optimal concentration in the semisolid agarose was 0.02%; higher concentrations of SDS caused DNA fiber dispersion.

In our previous studies, we used highly purified bovine pancreatic trypsin (EC.3.4.21.4) [41] for in-gel proteolysis. Its substrate specificity and pH dependence are optimal for digesting the DNA–protamine complex. Protamines are arginine-rich basic proteins, and trypsin specifically cleaves the carboxyl ends of lysine and arginine residues [41]. If sperm is embedded in agarose containing trypsin at pH 9.0, the DNA quickly diffuses. Since trypsin activity depends strictly on pH, it can be kept inactive at pH 4.7 until the gel solidifies and then reactivated by immersing the gel in cell-lytic reagents (pH 8). Commercially available twice-crystallized bovine trypsin is often contaminated with pancreatic DNases, so affinity chromatography using lima bean trypsin inhibitor-conjugated Sephacryl [42] is needed to remove autolyzed trypsin and DNases. Purified trypsin autolyzes readily and should be stored in solution at pH below 2.0. Although trypsin is ideal for in-gel proteolysis, it lacks versatility. Proteinase K [40], which has a chymotrypsin-like broad substrate specificity for aliphatic and aromatic amino acids, is active over a wide pH range; commercial preparations are available for DNA extraction from somatic cells. However, its substrate specificity makes it unsuitable for protamines [28]. Competitive dissociation of protamine–DNA complexes with DTT and SDS, followed by digestion of other nucleoproteins with proteinase K, allows DNA fibers to migrate via SCPFGE [28]. Overall, careful control of pH, temperature, moisture, antioxidants, and timing is essential to ensure reproducibility during simultaneous in-gel swelling and proteolysis.

### 5.4. Eligibility Criteria of Test Sperm for SCPFGE

We became acutely aware of the importance of eligibility criteria for test samples when investigating immunological infertility via anti-sperm antibodies (ASAs) [23]. During those studies, immunoglobulin G was partially purified from the female partner’s serum, and the localization of antigenic sites on PS and DS was compared using indirect immunofluorescence staining. PS was highly immunogenic for ASAs, but DS had lost its antigenicity [23]. Previous researchers did not consider the heterogeneity of human semen. Even in normozoospermic semen, DS comprises most of the sperm, and testing for ASAs in unseparated sperm can lead to false-negative results [23]. The goal of DNA fragmentation analysis is to detect early signs of damage in PS. However, once sperm are fixed, stained, embedded, or lysed, it becomes difficult to determine the original sperm type. The eligibility criteria for test specimens should be clearly defined and limited to PS. As shown in Section 4.1, SCSA also did not meet this eligibility requirement.

### 5.5. Low-Temperature Liquid Storage of Human Sperm

Motile sperm analyzed via 1D-SCPFGE immediately after preparation did not show noticeable differences in electrophoretic features compared to those analyzed after cryopreservation. Using 2D-SCPFGE, we observed unexpected physical damage to the DNA caused by ice crystals. When comparing with 2D-SCPFGE-0-150, the cryopreserved sample produced significantly more fibrous segments, but not granular segments, after the second electrophoresis run than fresh samples and those stored in low-temperature liquid form [28]. Currently, when using 2D-SCPFGE to detect naturally occurring DNA fragmentation, the artifactual fibrous segments caused by cryopreservation lead to false-positive results. If testing must be delayed, the previous study used an anti-freezing medium containing 50% propanediol for liquid storage [28]. Certain water-soluble, non-aqueous solvents such as ethylene glycol, propanediol, and glycerin are much better at stabilizing DNA sequences than water. We developed a modified low-temperature storage medium (0.5 mmol/L EDTA, 0.05% Triton X-100, 10 mmol/L ED in anhydrous ethylene glycol) to protect sequence integrity. ED is a scavenger of multiple reactive oxygen species (ROS) [43,44]. To reduce moisture content, the aqueous buffer containing sperm should comprise less than 5% of the storage medium. Although we confirmed the excellent protective performance of this formula for DNA, its practical use remains limited.

As described in Section 5.1, sperm adhere to the agarose-coated glass slide through hydroxyl–hydroxyl hydrogen bonding, and the two hydroxyl groups of ethylene glycol competitively inhibit this adhesion. A dilution of at least 1000-fold is necessary to permit sperm adherence. Currently, we tentatively recommend ethylene glycol as the preferred solvent for low-temperature liquid storage. We are working to identify an organic solvent that does not form hydrogen bonds. The sample stored in ethylene glycol is diluted in an appropriate aqueous medium containing 10 mmol/L ED and 0.5 mmol/L EDTA just before analysis.

### 5.6. Antioxidative Electrophoresis Buffer

Unlike proteins with a specific isoelectric point, anionic DNA moves toward the cathode regardless of the pH of the electrophoresis buffer. For example, its electrophoretic mobilities are similar in acetate-NaOH (pH 3.9), Tris-acetate (pH 8.2), and Na_2_CO_3_-NaHCO_3_ (pH 10.0) buffers. The pH range is limited by the tolerance of phosphodiester bonds to acid and alkaline hydrolysis. Electrolysis at the cathode produces singlet oxygen. Electrophoresis buffers containing ED become brown near the cathode over time due to ROS-induced oxidation, and dissolved oxygen also causes ED discoloration even when no current is applied. The addition of ascorbic acid (AA) prevents ED discoloration, highlighting the importance of water-soluble ROS scavengers in DNA electrophoresis. The antioxidative activity of AA targets ROS such as superoxide anions, hydrogen peroxide, and singlet oxygen, as well as hydroxyl radicals [45]. ED also scavenges multiple free radicals [43,45,46], especially hydroxyl radicals [47]. While ED is soluble in lower alcohols and DMSO and remains relatively stable in these non-aqueous solvents, it is poorly soluble and easily oxidized in water. Since the cathode generates singlet oxygen, we used AA as an inexpensive radical scavenger in the electrophoresis buffer. As discussed in Section 6.1, the pro-oxidant action of AA in the presence of transition metals like Fe and Cu must be considered [48,49], and EDTA inhibits this process. Because EDTA is a tetramer of acetic acid, we use salt-free EDTA as a substitute for acetic acid. AA powder, equivalent to 10 mmol/L, is dissolved in the basic solution (20 mmol/L Tris, 1.0 mmol/L EDTA, pH 9.2) just prior to use, resulting in a solution with a pH of 8.0.

### 5.7. DNA Staining with Fluorescent Dye and Brightening of Digital Images

We used an epifluorescence microscope with a green filter (Axio Imager A1; Carl Zeiss Microimaging, Jena, Germany) to observe the electrophoretic profile and recorded still images with a high-resolution charge-coupled device camera (AxioCam HRC; Carl Zeiss Microimaging) at a 0.4 s exposure. After electrophoresis, the gel film (60 μL) was equilibrated with an antioxidative electrophoresis buffer, and an equal volume of staining solution (X3000 SYBR Gold (Thermo Fisher Scientific, Waltham, MA, USA), 0.2 mol/L ED in DMSO) was added for 5 min. Excess dye was removed using blotting paper. After staining, the gel was equilibrated with a solution containing 0.1 mol/L ED, 5.0 mmol/L AA, and 0.5 mmol/L EDTA in 50% DMSO.

Photobleaching under the fluorescence microscope involves degradation of the dye molecule by ROS [50]. SYBR Gold is one of the most sensitive fluorescence dyes for double-stranded DNA, with high photobleaching tolerance. When observing the naked DNA fibers dyed with SYBR Gold under a fluorescence microscope, those suspended in water were extremely fragile. The synergistic effects of shearing force from Brownian motion and exposure to blue light visibly cleaved the sequence into granular segments. After SCPFGE, the elongated fibers fixed in the agarose network were quiescent, whereas prolonged exposure to the excitation with blue light caused DNA fragmentation [21]. If prolonged exposure cleaves the fibrous segments within the ROI, the artifactually produced granular segments lead to false-positive results. Thus, dark images resulting from short exposure times had to be brightened; for this, we used pseudo-exposure in Photoshop CS6 (Adobe, San Jose, CA, USA). At present, the absence of visible segments in the ROI after image enhancement is deemed sufficient to label the DNA as sequentially intact. Direct observation through an ocular lens is more sensitive than observation via a digital camera. When we determined the rate of DNA fragmentation by eye, we counted more than 100 sperm. We discuss the definition of “intact” sperm in DNA fragmentation analyses in Section 7.

The combination of 5.0 mmol/L AA and 0.1 mol/L ED in 50% DMSO shows strong anti-DNA fragmentation activity under light excitation. Generally, DNA fluorescent dyes contain cationic and hydrophobic regions; the former, usually tertiary amines, bind to the phosphates in the DNA backbone, while the latter produce fluorescence through intercalation into the major and/or minor grooves of DNA. When SYBR Gold is dissolved in lower alcohols, the hydrophobic environment suppresses intercalation, and DNA fibers are deformed due to dehydration. Currently, DMSO is the ideal solvent for SYBR Gold staining and the anti-DNA fragmentation activity of AA and ED. Additionally, DMSO acts as a free radical scavenger, particularly preventing DNA nicking caused by ionizing radiation or hydroxyl radicals generated by iron/hydrogen peroxide [51]. These agents do not deform DNA fibers, and the anti-DNA fragmentation effect persists during extended exposure.

When the swollen DNA mass (Figure 4A) is stained with the previously mentioned formula before 1D-SCPFGE, the long-chain fibers are rarely discharged from the origin. The electrophoretic mobility of DNA depends on the anionic phosphates in the backbone. The lack of discharge may result from a reduction in negative charge caused by the binding of many SYBR Gold molecules to the sequence. DMSO helps the dye to intercalate, which enhances fluorescence. The use of the current formula is limited to post-electrophoretic staining.

### 5.8. Accuracy Control of SCPFGE

As is well known, agarose forms a thermoplastic, large-pore gel through cross-linking of polysaccharide chains by hydrogen bonds. Commercially available agarose preparations vary in melting and gelling temperatures as well as mechanical strength, depending on the density of hydrogen-bond cross-links. SGE in agarose is the most common technique in molecular biology, and researchers often overlook the diversity among agarose types. The key technical skill in SCPFGE is selecting the appropriate agarose preparation. Larger pores are needed for electro-elongation of naked DNA fibers, which requires sparse cross-links; such gels have low melting points and weaker mechanical strength. The gel is prepared at the minimum gelation concentration to maximize pore size. For example, when we determined the optimal concentration of agarose GB for SCPFGE, we found that a 0.38% gel supported fiber elongation, whereas fibers were considerably shorter in 0.55% gels, and 0.29% solutions did not gel. In-gel swelling causes the densely packed DNA to untangle (Figure 1G and Figure 4A), leading to increased release of long DNA chains during the first electrophoresis and less backward discharge in the second (Figure 4B,C). If water evaporates during swelling and proteolysis, these effects are negated, as shown in Figure 1H; pore shrinkage results in a higher number of fibers discharging obliquely backward in the second electrophoresis. Therefore, detecting early signs of natural fragmentation is technically challenging, and the procedures outlined in this review were optimized for 0.4% agarose GB with careful moisture control. Even at the same concentration, high cross-linking preparations may yield false negatives. When using different preparations, precise control with PS is essential for reproducibility. In particular, the temperature during in-gel swelling and the agarose concentration should be optimized to produce electrophoretic profiles similar to those obtained with 0.38% agarose GB.

## 6. DNA Cleavage Analyses

2D-SCPFGE has been developed as a diagnostic tool to detect early signs of naturally occurring DNA fragmentation. All efforts described in Section 3, Section 4 and Section 5 focused not only on increasing sensitivity but also on preventing artifactual fragmentation during in vitro processing.

**Figure 5:**
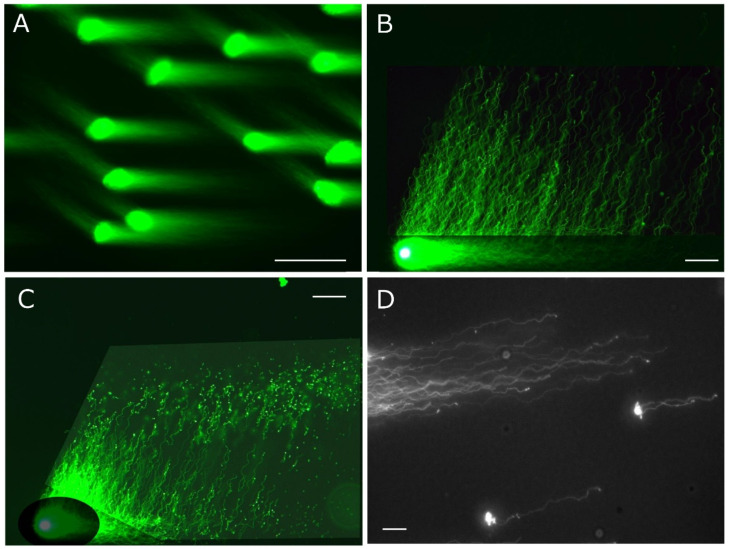
Analyses of enzymatic DNA cleavage and prokaryotic DNA. (**A**) The integrity of DNA used as the substrate was validated using 2D-SCPFGE-0-150. (**B**) 2D-SCPFGE-0-75 (3/3 min), where the long-chain fibers were aligned without overlapping. (**C**) The gel was removed from the rack after the first electrophoresis. The reaction mixture (27.7 U EcoR1/mL in 50 μL of 50 mmol/L Tris-HCl, pH 8.0, 100 mmol/L NaCl, 10 mmol/L MgCl2, and 1.0 mmol/L DTT) was applied and then incubated at 37 °C for 30 min in a moisture chamber. Subsequently, the slides were placed on the rack and rotated 75° for the second electrophoresis. The scale bars in (**A**–**C**) each represent 50 μm. (**D**) The profile of prokaryotic DNA in 1D-SCPFGE. Some bacteria contaminated during culture medium preparation were added to PS. The photograph was taken in monochrome to enhance the weak fluorescence. The scale bar represents 10 μm.

To date, we have examined artificial cleavage of DNA by means of heat denaturation [26], alkaline hydrolysis (Section 4.2) [22], Brownian motion (Section 5.7) [21], excitation by blue light (Section 5.7) [21], hydroxyl radicals produced via the Fenton reaction (Section 6.1) [48], DNase I [26], and a restriction endonuclease (EcoRI; Section 6.2) [28]. We also noted that ice crystals generate fine mechanical damage that is detectable only via 2D-SCPFGE (Section 5.5) [28]. Naked long-chain fibers are extremely sensitive to chemical and physical factors; they are readily cleaved down to granular segments, mimicking end-stage fragmentation. Hence, to avoid artifactual cleavage, careful attention (see Section 3, Section 4 and Section 5) is indispensable to observe the early symptoms of naturally occurring DNA fragmentation. In practical use, 1D-SCPFGE is a sufficiently sensitive tool for detecting chemical cleavages, in which case the techniques described in Section 3, Section 4 and Section 5 are optional. As the substrate for cleavage testing, the DNA integrity of the sperm population should be confirmed using 1D- or 2D-SCPFGE, depending on the required sensitivity. Figure 5A shows the massed profiles of 2D-SCPFGE-0-150 for use in the substrate. All the sperm were deemed to be intact. The sample was preserved in the low-temperature liquid storage medium.

### 6.1. Dose-Dependent DNA Cleavage by Hydroxyl Radicals

AA produces hydroxyl radicals in the presence of transition metals through the Fenton reaction [45]. 1D-SCPFGE showed that a single dose of sodium ascorbate (AA-Na) administered to sperm with the plasma membrane extracted using Triton X-100 caused DNA fragmentation in a dose-dependent manner [48]. Just 1.0 μmol/L AA-Na created fibers of various lengths, including long-chain fibers. As the AA-Na concentration increased, the fibers became shorter, and more granular segments appeared. Eventually, nearly all fibers degraded into granular segments, and the mass at the origin decreased at 100 μmol/L AA-Na. This degradation was fully prevented by 0.5 mmol/L EDTA 2Na. A single dose of AA-Na did not cause cleavage of naked DNA fibers, but the addition of 0.5 mmol/L CuSO4 enhanced the effect. The combined use of 1.0 mmol/L ED completely blocked the activity of both AA-Na and CuSO4 [48].

When discussing the impact of ROS on cellular functions, including DNA integrity, we must consider time, distance, and shielding, as ROS are short-lived and only oxidize a few molecules at their formation site. Exogenous AA reacts with transitional metals in the nucleus of plasma-membrane-extracted sperm, while swimming sperm with an intact plasma membrane are protected from extracellular AA [48]. Since AA does not act as a pro-oxidant in the presence of EDTA, we used AA and EDTA as counter-ions of Tris to create an antioxidative electrophoresis buffer (see Section 5.6).

### 6.2. Convenience of 2D-SPFGE for Analyses of Enzymatic DNA Cleavage

In macroscopic PFGE, the activity of proteinase K is terminated with phenylmethylsulfonyl fluoride prior to the application of restriction endonucleases. As proteinase K is washed out from the gel film during the first electrophoresis in 2D-SCPFGE, enzymatic DNA cleavage can be observed without the need to inhibit proteinase K. Naked DNA fibers are preferable as the enzymatic substrate to avoid steric hindrance caused by the tightly packed DNA–nucleoproteins complex [28]. For the control, a bundle of long-chain fibers is elongated during the first electrophoresis of 2D-SCPFGE-0-75 (3/3 min). In the second electrophoresis, those fibers are elongated obliquely forward and aligned with one another without overlapping (Figure 5B). For the test sample, the glass slide is ejected after the first electrophoresis, and the enzyme solution is mounted on the gel film. The segments are elongated obliquely forward, and the first electrophoresis served as proof that they were newly generated from the long-chain fibers. We previously reported the dose-dependent action of EcoR1 [28]. When treated with 27.7 U/mL EcoR1, almost all the long-chain fibers elongated during the first electrophoresis were cleaved into segments of diverse sizes (Figure 5C). As the concentration of endonuclease was increased, the size of the segment fibers was cleaved into shorter fragments. For DNA-cleaving enzymes that require divalent cations, EDTA in the electrophoresis buffer must be trapped. For instance, when the action of DNase I is investigated, excess MgCl_2_ and CaCl_2_ may be included in the reaction buffer to chelate EDTA. Under those circumstances, DNase I digests all the long fibers into granular segments without preconditioning. In general, the electrophoresis buffer should be removed before examining enzymatic activity.

## 7. Preparation of Huge DNA Segments to Mimic Early-Stage Fragmentation for 2D-SCPFGE

The current 2D-SCPFGE protocol is the most sensitive method for visualizing single-nuclear DNA fibers. In theory, the longest segments come from chromosome 1 being cut in half; however, it is unclear whether such long segments can be separated using this protocol. To find its separation limit, a set of chromosomal-level DNA fibers is needed as calibration standards. We planned to use prokaryotic DNA fibers as standards for 2D-SCPFGE, but they were too short to serve as size markers for very large eukaryotic DNA segments (Figure 5D). These standards would need to be created through artificial cleavage of PS. Currently, no such process exists. Until such standards are available, 2D-SCPFGE will remain a semi-quantitative method for early-stage fragmentation analysis. We tentatively suggest 2D-SCPFGE as a fairly accurate way to determine DNA sequence order, and sperm without long DNA segments in the ROI after image processing may be considered “intact”.

## 8. Future Work—Telomeric Ends and Cut Ends

**Figure 6:**
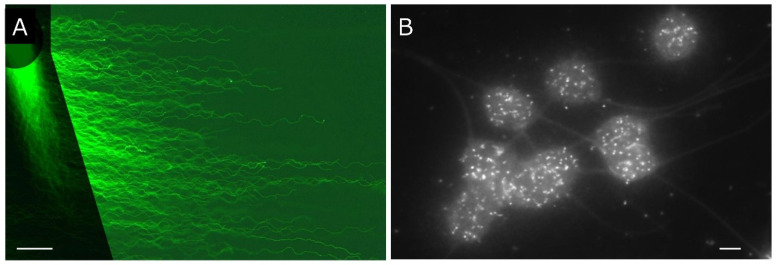
2D-SCPFGE-(-75)-0 proposes a new perspective on DNA fragmentation analysis. (**A**) Profile of a set of single-nuclear DNA fibers using 2D-SCPFGE-(-75)-0 (3/5 min). The scale bar represents 50 µm. (**B**) PS was adhered to a flat glass slide and swollen with 5.0 mmol/L DTT, pH 9.0, for 5 min. The repeat sequence of human telomeres (TTAGGG) was visualized according to the FISH protocol provided in the peptide nucleic acid (PNA) FISH Telomere/FITC kit (Dako Cytomation, Glostrup, Denmark). The scale bar represents 10 µm.

Although 2D-SCPFGE was developed to separate large DNA segments, it involves one or more angle rotations that produce various 2D alignments of DNA fibers [28]. We are exploring a new approach for DNA fragmentation analysis. Fluorescent in situ hybridization (FISH) allows mapping of specific DNA sequences in metaphase chromosomes and interphase nuclei. FISH on naked DNA fibers offers the highest resolution for mapping. The first step of our research is to align long-chain fibers without overlap. Figure 6A shows the profile of a set of single-nuclear DNA fibers created via 2D-SCPFGE-(-75)-0 (3/5 min). The elongated fibers were aligned laterally without overlapping. Figure 6B shows our initial telomeric FISH experiment. The repeat sequence of human telomeres [52] was visualized using peptide nucleic acid (PNA) FISH. PNA is a nucleobase oligomer where the entire backbone has been replaced by *N*-(2-aminoethyl) glycine units. It can identify specific DNA and RNA sequences by binding through strand invasion and forming a stable PNA/DNA/PNA triplex with a looped-out DNA strand [53]. The dispersed fluorescent signals indicate that the swollen DNA mass hybridized with the PNA telomere probe. The fluorescent signals at the fiber tips represent telomeric ends, while ends without signals are the cut ends (Figure 1I). Human sperm have 46 telomeric ends; the number of cut ends increases as chromosomes are cleaved into more segments. After aligning a set of single-nuclear long-chain fibers, we designated sperm with at least one cut end as damaged. The major technical challenge remains the solid-phase fixation and stabilization of these fibers. Since elongated naked DNA is very fragile in aqueous solution, it cannot withstand hybridization. We have found that certain non-aqueous solvents, such as ethylene glycol (Section 5.5) and DMSO (Section 5.7), effectively stabilize the sequences. DMSO is commonly used to lower the melting point of DNA, facilitating hybridization in FISH. Even if the DNA is somewhat cleaved during labeling, the position of the telomeric region can still be identified. This technique could evolve into a highly sensitive method for detecting early stages of DNA fragmentation.

## 9. Concluding Remarks on Basic and Clinical Studies

As illustrated in this review, quantifying naturally occurring early-stage fragmentation is technically challenging, but it is essential to establish detailed instructions to prevent false positives and negatives. We have built technical expertise for various steps of 2D-SCPFGE to improve its quantitative accuracy. Naked DNA that is tested after in-gel proteolysis can be used as a specimen; however, it is susceptible to ROS, so antioxidants are needed to prevent artifactual sequential damage in vitro. Surprisingly, cryopreservation also caused artifacts. Although the updated protocol for 2D-SCPFGE was refined from the previous one, it remains a semi-quantitative analysis method.

For many years, intraoperative sperm selection in ICSI primarily relied on gross morphology and motility observed under bright-field optics. We have developed several preoperative clinical tests besides 2D-SCPFGE: sperm-specific dye- and lectin-exclusion assays for assessing plasma and acrosomal membranes [24]; dye-retention assays for evaluating mitochondrial organelle membranes and endogenous ROS in the mitochondria [24]; and visualization of vacuoles in the sperm head [54,55]. Studies using these methods revealed that motile sperm often exhibited various impairments beyond DNA damage. Consequently, the criteria for selecting injectable sperm in clinical ICSI have become more complex, as such impairments cannot be detected with bright-field optics. To date, many cohort studies have shown that DNA damage is a significant factor in fertilization, post-implantation embryo development [12], and sperm-related congenital anomalies in ART [13]. However, these conclusions were based on conventional methods (see Section 4). The main goal of preoperative sperm examination is to determine which impairments or their combinations in motile sperm make them unsuitable for injection. This underscores the importance of a multifaceted approach. Cohort studies should include well-designed, multivariable analyses with careful consideration of confounding factors. Advancing technical skills—not only for 2D-SCPFGE but also for other methods—is an urgent priority, as it will allow us to revisit issues related to male infertility and DNA fragmentation.

## Data Availability

The datasets generated and analyzed in the present study are available from the corresponding author upon reasonable request.
